# A comparison of the operative outcomes of D1 and D2 gastrectomy performed at a single Western center with multiple surgeons: a retrospective analysis with propensity score matching

**DOI:** 10.1186/s12957-018-1422-6

**Published:** 2018-07-09

**Authors:** Susanna Lam, Elinor Tan, Audrey Menezes, David Martin, James Gallagher, David Storey, Charbel Sandroussi

**Affiliations:** The Upper Gastrointestinal Unit, Royal Prince Alfred Hospital, 50 Missenden Road, Sydney, NSW 2050 Australia

**Keywords:** Gastric cancer, Gastric adenocarcinoma, D1 and D2 lymphadenectomy, Lymphadenectomy, Lymph node dissection

## Abstract

**Background:**

There has been worldwide debate on lymphadenectomy for gastric cancer, with increasing consensus on performing an extended (D2) resection. There is a paucity of data in Australia. Our aim is to compare overall outcomes between a D1 and D2 lymphadenectomy for gastric cancer in a single specialist unit.

**Methods:**

We performed a retrospective analysis on patients who underwent a curative primary gastric resection for gastric adenocarcinoma between January 1996 and April 2016, primary outcomes included overall survival (OS) and disease-free survival (DFS). Propensity score matching (PSM) analysis was used to balance covariates between D1/D1+ and D2 groups. Kaplan-Meier survival curves of D1/D1+ versus D2 were constructed and evaluated using the log-rank test with subgroup analyses for pathological node (pN) status. Multiple Cox proportional hazards model was used to determine predictors of overall survival.

**Results:**

Two hundred four patients underwent a gastrectomy, 54 had D1/D1+, and 150 had a D2 lymphadenectomy. After PSM, there were 39 patients in each group, the 10-year OS for D1/D1+ was 52.1 and 76.2% for D2 (*p* = 0.008), and 10-year DFS was 35% for D1 and 58.1% for D2 (*p* = 0.058). Subgroup analysis showed that node-negative (N0) patients had improved 5-year OS for D2 (90.9%), compared to D1/D1+ (76.4%) (*p* = 0.028). There was no difference in operative mortality between the groups (D1 vs D2: 2 vs 0%, *p* = 0.314), nor in post-operative complications (*p* = 0.227). Multiple Cox analysis showed advanced tumor stage (stages III and IV), and lymphadenectomy type (D1) and the presence of postoperative complications were independent predictors of poor overall survival.

**Conclusions:**

D2 lymphadenectomy with spleen and pancreas preservation can be performed safely on patients with gastric adenocarcinoma. Significant improvement in overall survival is observed in patients with N0 disease who underwent D2 lymphadenectomy without increasing operative morbidity or mortality. This paper supports the notion of a global consensus for a D2 lymphadenectomy, particularly in the Western context.

## Background

Gastric cancer is the fifth most common malignancy and the third leading cause of cancer death worldwide [1]. In Australia, it is the ninth leading cause of cancer-related death [2]. Surgical resection of a primary tumor provides the best chance of cure for gastric cancer. However, the extent of lymph node resection for curative intent varies between surgeons and centers globally and within the Australian context. The lymph node status in gastric cancer is a key prognostic factor in patient survival [3, 4].

Historically, there has been controversy regarding the extent of lymph node dissection performed between Eastern and Western countries. East Asian countries [5] have consistently recommended the more extensive D2 lymph node dissection as standard care. Western nations have been cautious to adopt the D2 lymphadenectomy based on the short-term follow-up results of randomized control trials. The United Kingdom (UK) MRC-STO1 trial [6] and the Dutch DGCT trial [7] found no statistical difference in survival, but a significantly higher morbidity and mortality in the D2 group compared to D1. This was attributed to poor surgical technique compared to Eastern surgeons and the inclusion of standard distal pancreatectomy and splenectomy as part of the D2 lymphadenectomy. However, a 15-year follow-up of the same Dutch trial demonstrated that there was improved local regional recurrence and fewer gastric cancer-related deaths after a D2 dissection [8]. The Italian Gastric Cancer Study Group (IGCSG) did not show a significant difference in overall 5-year survival between D1 and D2 patients; however, a subgroup analysis indicated a trend towards improved survival for T2–T4 tumors and node-positive disease [9]. More recently, there is increasing consensus on a D2 lymphadenectomy with spleen and pancreas preservation becoming the standard of care, particularly in European centers.

In Australia, there is a relative lack of data on gastric cancer outcomes and more specifically on D1 and D2 lymphadenectomy. Some units have adopted selective criteria for patients undergoing D2 lymphadenectomy, with no significant difference in 5-year survival or mortality [10].

The aim of this study was to evaluate the operative outcomes of patients undergoing D1/D1+ and D2 gastrectomy for gastric adenocarcinoma.

## Methods

### Patient selection

A retrospective analysis was performed on 204 patients who underwent gastrectomy for gastric adenocarcinoma at our institution between January 1996 and April 2016. Patients were classified into two groups on the basis the type of lymphadenectomy performed: D2 lymphadenectomy (*n* = 150) and D1/D1+ lymphadenectomy (*n* = 54). Patients with prior gastric resection were not included. A standardized D1/D1+ or D2 lymphadenectomy with spleen/pancreas preservation was performed according to surgeon preference. D1/D1+ or D2 was performed as a standard preference for each surgeon, who are specialists trained in upper gastrointestinal surgery. There was no temporal difference between more D2 resections performed recently. Splenectomy and distal pancreatectomy were performed if there was direct tumor involvement.

### Operative procedure

According to the Japanese gastric cancer treatment guidelines 2014 (version 4), the Japanese Gastric Cancer Association (JGCA) defined the extent of lymphadenectomy according to the type of gastrectomy [5]. For total gastrectomy, lymph nodes dissected in D1 include nodes in stations 1 to 7; D1 plus (D1+) includes nodes in D1 stations plus 8a, 9, and 11p; D2 includes nodes in D1 stations plus 8a, 9, 10, 11p, 11d, and 12a. Station 10 lymph node dissection may be omitted [11]. For subtotal gastrectomy, distal gastrectomy includes lymph node dissection in D1 including nodes in stations 1, 3, 4sb, 4d, 5, 6 and 7; D1 plus (D1+) includes nodes in D1 station plus 8a and 9; and D2 includes nodes in D1 stations plus 8a, 9, 11p, and 12a.

### Data collection

Preoperative demographic data included age, gender, and body mass index (BMI). Pathological data included tumor location, depth of tumor (T), nodal status (N), tumor stage (TNM) stratified according to the American Joint Committee on Cancer (AJCC) 7th edition staging system [12], and tumor differentiation type (well, moderate, poor). Treatment data comprised of gastrectomy type (total, partial), lymphadenectomy type (D1/D1+, D2) [13], lymph node yield, and perioperative chemotherapy (neoadjuvant and/or adjuvant therapy). Postoperative data included 30-day mortality, postoperative complications graded according to the Clavien-Dindo classification [14], 5-year overall survival (OS), and 5-year disease-free survival (DFS).

### Propensity score analysis

Propensity score matching (PSM) analysis was performed. Patients undergoing D1/D1+ lymphadenectomy were matched to patients undergoing D2 lymphadenectomy based on similar estimated propensity scores. This is to balance covariates in the two groups to reduce selection bias [15]. Propensity scores are estimated using a logistic regression model that calculates the probability of a D1/D1+ or D2 assignment on observed baseline characteristics [16]. The variables included in the model were body mass index (BMI), tumor depth, tumor location, and perioperative chemotherapy. We used the nearest neighbor 1:1 propensity matching with matched without replacement method. To ensure suitable equivalents of matches made on propensity scores, a caliper width was imposed of 0.2 of the standard deviation of the logit of the propensity score [17]. Calculating the propensity score requires the inclusion of covariates that predict potential outcomes under each treatment arm, as well as covariates that predict treatment assignment, which are usually related [18, 19].

Only four parameters were used in the matching process as the overall patient numbers in this study are small, with 54 patients in the D1/D1+ group. If the sample size is too small for the propensity model to include all the variables of interest, then it is recommended to choose variables most strongly related to outcome [18, 20]. These variables are selected based on available evidence or previous literature. The parameters of tumor stage, tumor location, BMI, and perioperative chemotherapy were considered clinically relevant factors that have been shown in the literature to influence either outcome or extent of lymphadenectomy performed for gastrectomy patients.

Increased BMI is associated with higher death rates from all cancers compared to normal weight patients. In addition, there is increased risk with higher BMI and death from gastric cancer in men [21]. Comparison studies indicate Western patients are thought to be at higher risk of surgical morbidity and mortality, due in part to higher BMI than Japanese patients, who are leaner and generally undergo a standard D2 lymphadenectomy [22]. Tumor location affects patient survival [22], particularly tumors of the middle or upper third, with proximal stomach tumors having the worst prognosis [22]. Tumor (T) stage and depth of invasion are independent prognostic factors in gastric cancer [23, 24]. Increasing T stage is associated with poorer prognosis [25]. This was the rationale for including tumor factors that are strongly related to outcome in calculating propensity scores.

The use of chemotherapy in gastrectomy patients has been shown to affect survival. The United Kingdom Medical Research Council Adjuvant Gastric Infusional Chemotherapy (MAGIC) trial showed a higher likelihood of overall and progression-free survival in those undergoing perioperative chemotherapy [26]. According to the MAGIC protocol, tumors considered stage II were included in the eligibility criteria for chemotherapy. It was thought that including any perioperative chemotherapy as an adjusting variable in the PSM may reduce the possible selection or outcome bias associated with patients who were to undergo chemotherapy in our unit.

### Statistical analyses

Preoperative, pathological, treatment, and postoperative data were compared between groups. Results were expressed as the mean (standard deviation) and frequency (%). Categorical variables were compared using the chi-squared or Fisher’s exact test and continuous variables using the non-parametric Mann-Whitney *U* test or *t* test as appropriate. Kaplan-Meier survival curves were constructed and evaluated using the log-rank test. Survival time estimates were expressed as mean (95%CI). Sub-group survival analyses were conducted according to AJCC 7TH Edition pathological node (pN) status. Independent variables that predicted overall survival were assessed with the use of the Cox proportional hazards model that included variables with a univariate *p* value of less than 0.25. Backward elimination was applied to determine independent variables that significantly impact survival [27]. Statistical significance was set at *p* < 0.05. All statistical analyses were performed using the Statistical Package for the Social Science (SPSS) version 22.0 software.

## Results

### Patient characteristics

Two hundred four patients with gastric adenocarcinoma underwent primary gastric resection for gastric cancer over a 20-year period from 1996 to 2016 (Table 1). Of those patients, 54 underwent a D1/D1+ dissection, and 150 underwent a D2 dissection. In the unmatched group, the mean BMI in the D1/D1+ group was significantly higher and greater proportion of patients underwent a partial gastrectomy in the D1/D1+ group compared to patients in the D2 group (D1/D1+ versus D2: mean BMI, 26.7 vs 24.8 kg/m^2^, *p* = 0.049; partial gastrectomy, 81 vs 63%, *p* = 0.011). Lymph node yield was higher in the D2 group (D1/D1+ versus D2: mean lymph node yield, 15.7 vs 21.7, *p* = 0.001).

**Table 1:** Clinicopathological characteristics of patients undergoing gastrectomy with D1/D1+ or D2 lymphadenectomy

Variable		Before PSM (*n* = 204)					^a^After PSM (*n* = 78)				
		D1/D1+ (*n =* 54)		D2 (*n =* 150)			D1/D1+ (*n =* 39)		D2 (*n =* 39)		
		54		150			39		39		
		*n*	%	*n*	%	*p* ^b^	*n*	%	*n*	%	*p* ^b^
Age^d^	Mean (sd)	^c^67.1	^c^(11.7)	^c^65.5	^c^(12.75)	0.420	^c^67.4	^c^(11.5)	64.9	^c^(13.5)	0.387
Gender						0.604					1.000
	Male	35	(65%)	103	(69%)		27	(69%)	27	(69%)	
	Female	19	(35%)	47	(31%)		12	(31%)	12	(31%)	
BMI^e^	Mean (sd)	^c^26.7	^c^(5.46)	^c^24.8	^c^(5.58)	0.049*	^c^26.0	^c^(5.29)	^c^24.4	^c^(6.35)	0.219
Hemoglobin level						0.932					0.530
	≤ 100 g/L	7	(13%)	20	(13%)		5	(13%)	7	(18%)	
	> 100 g/L	47	(87%)	129	(86%)		34	(87%)	32	(82%)	
Tumor location						0.168					0.193
	Proximal	5	(9%)	30	(20%)		4	(10%)	6	(15%)	
	Middle	15	(28%)	31	(21%)		11	(28%)	17	(44%)	
	Distal	28	(52%)	79	(53%)		24	(62%)	16	(41%)	
TMN stage						0.119					0.280
	In situ	0	(0%)	4	(3%)		0	(0%)	4	(10%)	
	I	19	(35%)	44	(29%)		14	(36%)	16	(41%)	
	II	19	(35%)	49	(33%)		14	(36%)	11	(28%)	
	III	12	(22%)	48	(32%)		9	(23%)	7	(18%)	
	IV	4	(7%)	3	(2%)		2	(5%)	1	(3%)	
	Unknown	0	(0%)	2	(1%)		0	(0%)	0	(0%)	
T level						0.123					0.411
	T0	0	(0%)	4	(3%)		0	(0%)	2	(5%)	
	T1	15	(28%)	39	(26%)		11	(28%)	12	(31%)	
	T2	8	(15%)	18	(12%)		7	(18%)	7	(18%)	
	T3	22	(41%)	39	(26%)		15	(38%)	10	(26%)	
	T4	9	(17%)	48	(32%)		6	(15%)	6	(15%)	
	Unknown	0	(0%)	2	(1%)		0	(0%)	2	(5%)	
N category						0.831					0.523
	N0	26	(48%)	65	(43%)		18	(46%)	20	(51%)	
	N1	12	(22%)	35	(23%)		9	(23%)	12	(31%)	
	N2	7	(13%)	27	(18%)		5	(13%)	4	(10%)	
	N3	9	(17%)	23	(15%)		7	(18%)	3	(8%)	
Differentiation						0.389					0.422
	Well	1	(2%)	8	(5%)		1	(3%)	2	(5%)	
	Moderate	16	(30%)	52	(35%)		9	(23%)	14	(36%)	
	Poor	30	(56%)	65	(43%)		22	(56%)	15	(38%)	
	Unknown	7	(13%)	25	(17%)		7	(18%)	8	(21%)	
Chemotherapy						0.228					1.000
	Yes	30	(56%)	69	(46%)		22	(56%)	22	(56%)	
	No	24	(44%)	81	(54%)		17	(44%)	17	(44%)	
Gastrectomy type						0.011*					0.065
	Total	10	(19%)	56	(37%)		6	(15%)	13	(33%)	
	Partial	44	(81%)	94	(63%)		33	(85%)	26	(67%)	
Lymph node yield^e^	Mean (sd)	^c^15.7	^c^(10.1)	21.7	^c^(12.0)	0.001*	15.0	^c^(10.4)	20.3	^c^(11.5)	0.050*
Splenectomy +/− pancreatectomy						0.581					1.000
	No	53	(98%)	145	(97%)		38	(97%)	38	(97%)	
	Yes	1	(2%)	5	(3%)		1	(3%)	1	(3%)	

*p < 0.05

^a^Matched parameters—BMI, T-level, tumor location, chemotherapy

^b^Chi-square test

^c^Mean (sd)

^d^Mann-Whitney *U* test

^e^*t* test

After propensity score matching, 126 patients were excluded, resulting in two patient groups (*n* = 39 each) that had similar preoperative characteristics and tumor pathology (Table 1). Mean lymph node yield was still significantly different between both groups (D1/D1+ v D2: mean lymph node yield, 15.0 vs 20.3, *p* = 0.050).

### Survival

**Fig. 1:**
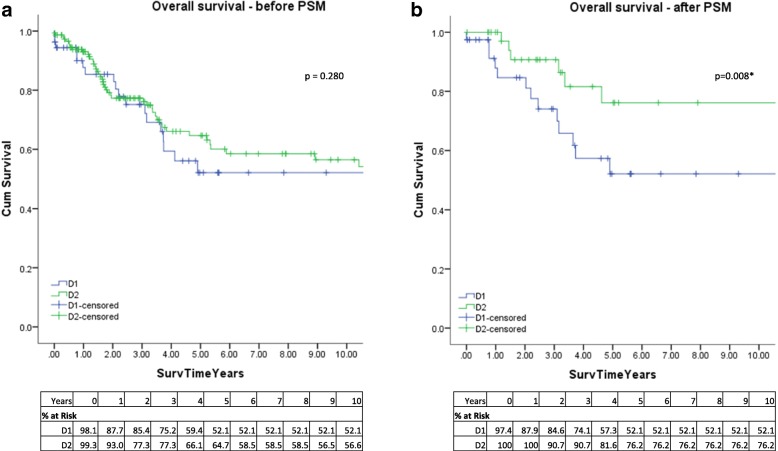
Kaplan-Meier overall survival for patients undergoing gastrectomy with D1/D1+ or D2 lymphadenectomy. **a** Before propensity score matching. **b** After propensity score matching (PSM). *p < 0.05

The median follow-up period was 4.6 years. Sixty-six deaths occurred during the follow-up (D1/D1+: *n* = 20 (37%), D2: *n* = 46 (30.7%). There were 68 patients with recurrence of disease with 17 (25%) occurring in the peritoneum. In the unmatched cohort, before PSM, the mean OS time for all patients was 11.40 years (95%CI 9.82–12.97). Mean OS time for D1/D1+ and D2 groups were 8.48 years (95%CI 5.80–11.16) and 11.97 years (95%CI 10.16–13.78), respectively. The 1-, 3-, 5-, and 10-year OS was 87.7, 75.2, 52.1, and 52.1% for the D1/D1+ group, and 93.0, 77.3, 64.7, and 56.6% in the D2 group respectively (Fig. 1a). There was no difference in OS (*p* = 0.280). After PSM, mean OS time for all patients was 11.97 years (95%CI 9.52–14.42). Mean OS for D1/D1+ and D2 groups were 7.14 years (95%CI 5.28–9.00) and 14.58 years (95%CI 11.66–17.86) years respectively. The 1-, 3-, 5-, and 10-year OS was 87.9, 74.1, 52.1, and 52.1% and 100, 90.7, 76.2, and 76.2% in the D1/D1+ and D2 group respectively. OS was significantly longer in patients in the D2 group (*p* = 0.008) (Fig. 1b).

**Fig. 2:**
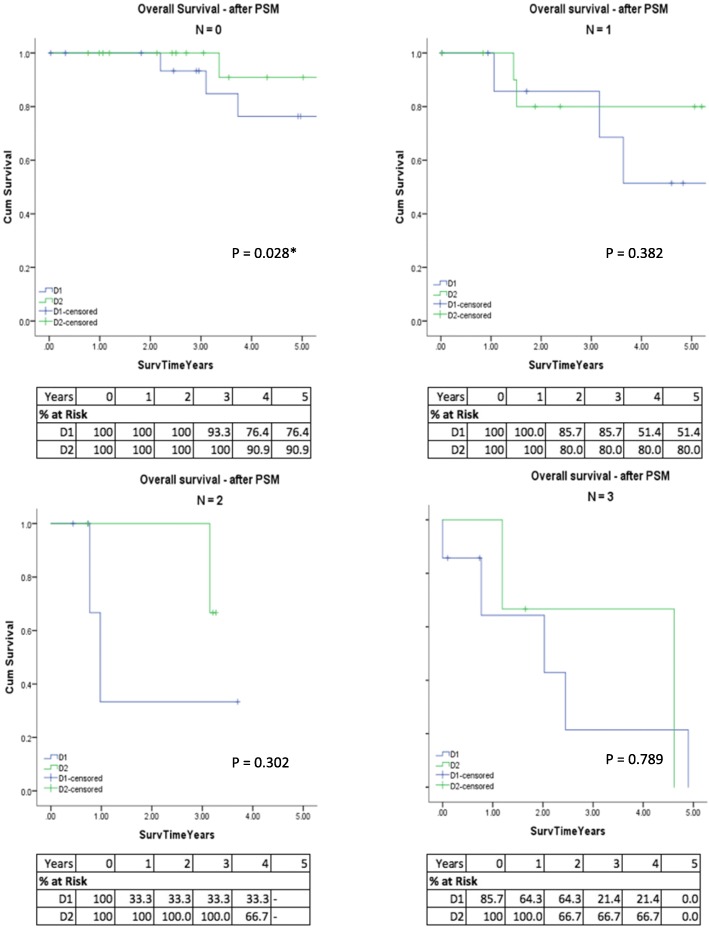
Kaplan-Meier overall survival for patients undergoing gastrectomy with D1/D1+ or D2 lymphadenectomy: After propensity score matching and stratified according to AJCC Nodal stages **a** N0, **b** N1, **c** N2, and **d** N3. *p < 0.05

When overall survival was stratified according to pathological nodal status, there was a significant difference in OS for D2 dissection for patients with N0 disease (no regional metastasis) where mean survival time was 9.47 years (95%CI 7.15–11.78) in the D1/D1+ group and 15.90 years (95%CI 12.14–19.66) in the D2 group and 5-year OS was 76.4% (D1/D1+) compared to 90.9% (D2) (*p* = 0.028) (Fig. 2).

**Fig. 3:**
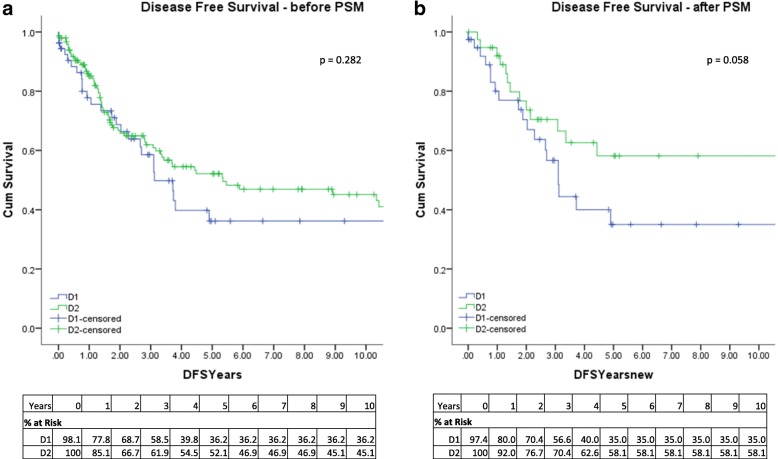
Kaplan-Meier disease-free survival for patients undergoing gastrectomy with D1/D1+ or D2 lymphadenectomy. **a** Before propensity score matching. **b** After propensity score matching (PSM)

There was no statistically significant difference in DFS between D1/D1+ and D2 groups. In unmatched cohorts, the 10-year DFS was 36.2% for D1/D1+ and 45.1% for D2 (*p* = 0.282), after PSM, the 10-year DFS was 35% for D1/D1+ and 58.1% for D2 (*p* = 0.058) (Fig. 3).

### Univariate analysis of complications and 30-day mortality

Overall in the unmatched cohort, there were 5 patients who suffered mortality within 30 days, with no significant difference between D1/D1+ 6% vs D2 1% (*p* = 0.085) (Table 2). There was no difference in mortality after PSM between D1/D1+ (2%) and D2 (0%) (*p* = 0.314). In univariate analysis of factors associated with 30-day mortality, total gastrectomy was a significant variable; however, this association was not present after PSM (Table 3).

**Table 2:** Postoperative outcomes of patients undergoing gastrectomy with D1/D1+ or D2 lymphadenectomy

Variable		Before PSM (*n =* 204)					^a^After PSM (*n =* 78)				
		D1/D1+ (*n =* 54)		D2 (*n =* 150)			D1/D1+ (*n =* 39)		D2 (*n =* 39)		
		*n*	%	*n*	%	*p* ^b^	*n*	%	*n*	%	*p* ^b^
Clavien-Dindo classification						0.454					0.227
	I	7	(13%)	31	(21%)		3	(7%)	10	(24%)	
	II	15	(28%)	50	(33%)		9	(21%)	11	(26%)	
	III	3	(6%)	7	(5%)		3	(7%)	1	(2%)	
	IV	2	(4%)	4	(3%)		1	(2%)	1	(2%)	
	V	3	(6%)	2	(1%)		1	(2%)	0	(0%)	
30-day mortality	Yes	3	(6%)	2	(1%)	0.085	1	(2%)	0	(0%)	0.314
	No	51	(94%)	148	(99%)		41	(98%)	42	(100%)	

^a^Matched parameters—BMI, T-level, tumor location, chemotherapy

^b^Chi-square test

**Table 3:** Factors associated with postoperative mortality and morbidity for patients undergoing gastrectomy with D1/D1+ or D2 lymphadenectomy

Variables	Before PSM (*n =* 204)							After PSM (*n =* 78)						
	No. of patients	^a^Complications			30-day mortality			No. of patients	^a^Complications			30-day mortality		
		*n*	%	*p*	*n*	%	*p*		*n*	%	*p*	*n*	%	*p*
Age				0.008*			0.557				0.006*			0.265
< 70	108	57	(53%)		2	(2%)		43	16	(37%)		0	(0%)	
≥ 70	96	68	(71%)		3	(3%)		35	24	(69%)		1	(3%)	
Gender				0.892			0.117				0.624			0.502
Male	138	85	(62%)		5	(4%)		54	28	(52%)		1	(2%)	
Female	66	40	(61%)		0	(0%)		24	11	(46%)		0	(0%)	
BMI				0.502			0.228				0.784			0.595
≥ 30	29	15	(52%)		1	(3%)		17	8	(47%)		0	(0%)	
< 30	135	79	(59%)		1	(1%)		61	31	(51%)		1	(2%)	
Hemoglobin level				0.830			0.075				0.530			0.688
≤ 100	25	17	(68%)		2	(8%)		12	7	(58%)		0	(0%)	
> 100	173	107	(62%)		3	(2%)		66	31	(47%)		1	(2%)	
Tumor location				0.009*			0.406				0.070			0.302
Proximal, middle	81	57	(70%)		2	(2%)		38	23	(61%)		1	(3%)	
Distal	107	55	(51%)		1	(1%)		40	16	(40%)		0	(0%)	
Tumor stage (TNM)				0.145			0.768				0.425			0.087
III, IV	67	46	(69%)		2	(3%)		19	11	(58%)		1	(5%)	
I, II	131	76	(58%)		3	(2%)		55	26	(47%)		0	(0%)	
Chemotherapy				0.849			0.699				1.000			0.252
Yes	99	60	(61%)		2	(2%)		34	17	(50%)		0	(0%)	
No	105	65	(62%)		3	(3%)		44	22	(50%)		1	(2%)	
Gastrectomy type				0.020*			0.021*				0.018*			0.076
Total	66	48	(73%)		4	(6%)		19	14	(74%)		1	(5%)	
Partial	138	77	(56%)		1	(1%)		59	25	(42%)		0	(0%)	
Lymphadenectomy type				0.314			0.085				0.113			0.314
D1/D1+	54	30	(56%)		3	(6%)		39	16	(41%)		1	(3%)	
D2	150	95	(63%)		2	(1%)		39	23	(59%)		0	(0%)	
Splenectomy +/− pancreatectomy				0.783			0.694				1.000			0.870
Yes	6	4	(67%)		0	(0%)		2	1	(50%)		0	(0%)	
No	198	121	(61%)		5	(3%)		76	38	(50%)		1	(1%)	

*p < 0.05

^a^Complications is defined as Clavien-Dindo grades I, II, III, and IV

All complications were recorded for D1/D1+ and D2 groups, and analyses were performed for matched and unmatched cohorts. For both cohorts, D2 patients had more Clavien-Dindo grade I and II complications compared to D1/D1+ (Table 2.) and there was no statistically significant difference in the rate of complications between unmatched (*p* = 0.454) nor PSM cohorts (*p* = 0.227) (Table 2.)

In unmatched cohorts, univariate analysis showed older age (> 70 yr.) (*p* = 0.008), proximal/middle tumor location (*p* = 0.009), and total gastrectomy (*p* = 0.020) were associated with postoperative complications and mortality. After PSM, advanced age (> 70 yr.) (*p* = 0.006) and total gastrectomy (*p* = 0.018) were also associated with post-surgical complications (Table 3).

### Cox proportional hazards regression analysis

In the unmatched cohort, total gastrectomy (*p* = 0.019) stage III /IV tumor (*p* = 0.000) and the presence of overall complications (Clavien-Dindo ≥ grade II) (*p* = 0.000) were poor prognostic factors. After adjusting for confounding effects in the multivariable analysis, stage III/IV tumors (HR 3.519 (95%CI 2.067–5.993), *p* = 0.000) and the presence of overall complications (Clavien-Dindo ≥ grade II) (HR 2.971 (95%CI 1.714–5.149), *p* = 0.000) were predictors of mortality. After PSM, the univariable analysis showed D1/D1+ lymphadenectomy (*p* = 0.013), total gastrectomy type, (0.034), stage III/IV tumors (*p* = 0.000), and overall complications (*p* = 0.001) were associated with mortality. Multivariable Cox regression analysis identified D1/D1+ (HR 4.353 (95%CI 1.511–12.514), *p* = 0.006), stage III/IV tumors (HR 4.218 (95%CI 1.560–11.407), *p* = 0.005), and overall complications (Clavien-Dindo > grade II) (HR 3.849 (95%CI 1.342–11.040), *p* = 0.012) as independent predictors of mortality. Results from univariable and multivariable analyses are summarized in Table 4.

**Table 4:** Cox proportional hazards model on prognostic factors for patients undergoing gastrectomy with D1/D1+ or D2 lymphadenectomy

		Univariable Cox regression analysis			Multivariable Cox regression analysis		
Parameters		HR	95%CI	*p*	HR	95%CI	*p*
Before PSM							
Age	≥ 70/< 70	1.172	0.721–1.907	0.521			
Gender	F/M	1.045	0.622–1.756	0.867			
BMI	< 30/≥30	1.138	0.673–1.924	0.629			
Hemoglobin level	≤ 100/> 100	1.682	0.854–3.314	0.133			
Tumor location	Proximal, middle/Distal	1.608	0.948–2.728	0.078			
TNM stage (AJCC classification)	III, IV/ I, II	3.925	2.402–6.414	0.000*	3.519	2.067–5.993	0.000*
Chemotherapy	Yes/No	1.205	0.740–1.963	0.453			
Lymphadenectomy type	D1/D1+/D2	1.338	0.787–2.272	0.282			
Gastrectomy type	Total/Partial	1.803	1.102–2.949	0.019*			
^a^Overall complication	Yes/No	3.169	1.895–5.298	0.000*	2.971	1.714–5.149	0.000*
After PSM							
Age	≥ 70/< 70	1.666	0.714–3.881	0.237			
Gender	M/F	1.069	0.416–2.744	0.890			
BMI	< 30/≥ 30	3.463	0.808–14.840	0.094			
Hemoglobin level	≤ 100/> 100	1.596	0.542–4.701	0.396			
Tumor location	Proximal, middle/Distal	1.305	0.572–2.981	0.527			
TNM stage (AJCC classification)	III, IV/ I, II	6.627	2.640–16.638	0.000*	4.218	1.560–11.407	0.005*
Chemotherapy	Yes/No	1.318	0.573–3.034	0.516			
Lymphadenectomy type	D1/D1+/D2	3.490	1.308–9.311	0.013*	4.353	1.511–12.541	0.006*
Gastrectomy type	Total/Partial	2.577	1.074–6.184	0.034*	2.883	0.846–9.829	0.091
^a^Overall complication	Yes/No	4.480	1.901–10.560	0.001*	3.849	1.342–11.040	0.012*

*p < 0.05

^a^Overall complication is defined as Clavien-Dindo ≥ grade II

## Discussion

There is now an increasing global consensus on performing a D2 lymphadenectomy for gastric cancer due to long-term results of large Western studies in conjunction with Eastern data [8, 28, 29]. This retrospective analysis of a single low to intermediate volume [30] specialist Western center indicates that a D2 lymphadenectomy can be performed safely, with excellent outcomes.

We performed direct comparison between D1/D1+ lymphadenectomy and D2 lymphadenectomy with regression analysis and also performed propensity score matching between the two groups in order to reduce selection bias. Ten-year overall survival for unmatched D1/D1+ was 52.1% and D2 was 56.6% (*p* = 0.280). After propensity score matching, OS was 52.1% for D1/D1+ and 76.2% for D2 which was significantly higher (*p* = 0.008) (Fig. 1.)

Our results showed improved long-term survival outcomes for D2 lymphadenectomy compared to results reported in Western literature and approaches those reported in Eastern studies. In a meta-analysis of four RCTs, the 5-year OS for D2 resections was 47% [31]. Specifically, the Japanese report 5-year OS of 69.2% and 10-year OS of approximately 60% for D2 resections [32]. The Italian IGCSG report 5-year OS of 64.2% (D2) [9] while the Dutch DGCT trial showed an 11-year OS of 35% for D2 groups (*p* = 0.53) [7] and subsequent 15-year OS 29% for D2 [8]. However surgical expertise and the inclusion of distal pancreatectomy and splenectomy were confounding factors affecting the results of the Dutch trial.

When stratifying the matched cohort according to nodal status, those with N0 status had higher 5-year OS, and there was a survival benefit for the D2 group (90.9%) compared with the D1/D1+ group (76.4%) (*p* = 0.028) (Fig. 2). A similar paradoxical finding was reported in a different study, whereby patients with clinically node-negative disease had a survival benefit with extended D2 plus para aortic lymph node dissection and may be a result of statistical bias [32]. Other studies have found a benefit for D2 dissection in N2 disease [7]. These findings suggest that an adequate lymph node dissection is important for adequate staging, and there are other poor prognostic indicators present in patients with node-negative disease [33]. Another explanation may be the presence of occult disease or micro metastatic disease in what is histopathologically described as node negative. Studies have shown that occult lymph node metastasis correlates with poor prognosis in node-negative disease and that a D2 resection may be beneficial in patients with node-negative occult disease detected by molecular techniques [34]. Nodal status is determined by histopathological assessment by pathologists, rather than surgeons at our unit, which may affect the accuracy of pathological reporting. Consequently, a thorough lymph node dissection allows capturing of more lymph nodes, in addition to prognostication.

Overall, 10-year DFS for the unmatched cohort was 36.2% for D1/D1+ and 45.1% for D2 (*p* = 0.282), after PSM, the 10-year DFS was 35% for D1/D1+ and 58.1% for D2 (*p* = 0.058) (Fig. 1). There was no significant difference between D1/D1+ and D2 in terms of recurrence. Our results are more consistent with long-term data obtained in Western studies, whereby the Dutch trial at 11 years reported risk of relapse was 65% for D2 [7] and 15 years DFS was 28% for D2 [8].

In this study, the overall 30-day mortality was 2.4% (unmatched) and 1.2% (after PSM), which is comparable to other units. The 2016 National Oesophago-Gastric Cancer Audit of England and Wales reports 2.2% in-hospital mortality and 30-day mortality rates range between 4.5 and 1.9% [35]. The D2 operative mortality was 1% in the unmatched cohort, consistent with other units [9, 32, 36].

The overall postoperative complication rate was 61% (unmatched) and 51% (after PSM) (Table 2). The majority were minor complications (Clavien-Dindo grades I and II). With respect to postoperative complications and 30-day mortality, there were no differences between the D1/D1+ and D2 groups for both matched and unmatched cohorts.

Factors associated with complications Clavien-Dindo grades I to IV, and post-operative death (Clavien-Dindo grade V, or 30-day mortality) are listed in Table 3. The tumor location between D1/D1+ and D2 groups were not significantly different (Table 1). In unmatched cohorts, there was an association with tumor location and complications (*p* = 0.009), this was no longer present after PSM (*p* = 0.07). Total gastrectomy was associated with postoperative complications in both matched and unmatched cohorts (before PSM, *p* = 0.020; after PSM, *p* = 0.018), which has been shown in other studies [7, 37]. Total gastrectomy was not associated with 30-day mortality after propensity score matching (*p* = 0.076), suggesting the influence of confounding factors in the unmatched cohort in which there was an association (*p* = 0.021) (Table 3).

Patients over the age of 70 years have increased morbidity and mortality associated with gastrectomy in Western studies [7]. In this study, advanced age (≥ 70 years) was associated with postoperative complications (before PSM, *p* = 0.008; after PSM, *p* = 0.006); however, these were mostly Clavien-Dindo grade I and II complications. Older age was not associated with 30-day mortality (Table 3). These findings highlight the importance of postoperative strategies required to optimize positive outcomes in the elderly to ensure safe delivery of surgical treatment [38].

Body Mass Index was not associated with post-operative morbidity or mortality. The mean BMI was significantly higher in the unmatched cohort between the D1/D1+ (26 kg/m^2^) vs D2 group (24 kg/m^2^) (*p* = 0.049) (Table 1), there was no difference in BMI after PSM. The average BMI in our patient cohort is similar to other Western units [10] and is higher than those reported in Japanese studies, where the median BMI is 23 kg/m^2^ [11].

Pancreaticosplenectomy was not routinely performed as part of the D2 lymphadenectomy and subsequently not associated with the post-operative complications or 30-day mortality. This is supported by other studies, whereby a D2 lymphadenectomy with spleen preservation avoids operative morbidity without affecting survival [11]. The Japanese have reported that no. 10 lymph nodes are often left untouched and can be dissected if judged easily removable in lean patients. Western studies have also reported that a modified D2 gastrectomy (with splenic preservation) is associated with improved survival, particularly in stage III gastric cancer [39].

Some surgeons who are proponents of D1/D1+ prefer to do so in Western patients who often have a higher BMI, arguing that a D2 is technically easier in slimmer, Eastern patients. The results for our cohort, with BMI < 30 kg/m^2^, indicate that an extended lymph node dissection with spleen and pancreas preservation can be performed in the Western cohort with no added morbidity.

The use of perioperative chemotherapy in the management of gastric cancer has been a major development over the past decade, with increased survival in perioperative chemotherapy versus surgery alone [26]. The number of patients receiving perioperative chemotherapy in this unit has increased over time, reflecting this change. The intention was for patients undergoing perioperative chemotherapy to follow the MAGIC protocol, with three cycles of epirubicin, cisplatin, and flurouracil (ECF) preoperatively and postoperatively [26]. The number of patients receiving chemotherapy did not differ between the groups. While it is recognized that receiving neoadjuvant chemotherapy would be more relevant to the perioperative complications and 30-day mortality than adjuvant therapy, perioperative chemotherapy usage was not associated with post-operative morbidity or mortality in our unit.

The results of our morbidity and mortality are similar to those reported in the literature, thus suggesting that identification of high-risk factors may allow more rational patient selection or systemic therapy [40]. In assessing prognostic factors for patients undergoing gastrectomy with D1/D1+ or D2 lymphadenectomy, univariate analysis demonstrated that patients with higher TNM stage (stages III and IV), a local lymphadenectomy (D1/D1+), total gastrectomy, and postoperative complications were significantly associated with poor prognosis. Furthermore, multivariate analysis showed that a higher TNM stage (stages III and IV), a D1/D1+ lymphadenectomy, and the presence of postoperative complications were prognostic factors (Table 4). This is similar to other Western studies which have shown higher TNM stage and lymphadenectomy to be prognostic indicators [6, 39].

Our results indicate that D2 lymphadenectomy with spleen/pancreas preservation is not associated with poor short-term and long-term outcomes whereas a D1/D1+ dissection was one of the factors that predicted poor overall survival in our unit. This finding is comparable to results of a recent meta-analysis [41] that pooled data from the UK MRC-STO1 trial [6], Dutch DGCT trial [8], and Taiwan trial [42] revealing a combined overall survival benefit in D2 lymphadenectomy with spleen and/or pancreas preservation.

### Limitations

This study is limited by its retrospective nature with possible selection and information bias. This data represented the experience of a single tertiary academic referral center over two decades and is subject to referral bias. In addition, the number of patients included in this analysis was relatively small resulting in a low powered study; however, this is considered a large series from a single Western center in Australia.

Furthermore, it is difficult to adequately account for the surgical techniques employed by the operators including contamination and compliance, which has been an issue in many surgical studies [9]. While the lymph node yield was significantly different between the D1/D1+ group and the D2 group in unmatched and propensity score matched groups, the exact nodal stations dissected by each surgeon is difficult to audit.

In comparing patients that underwent a D1/D1+ lymphadenectomy versus the D2 lymphadenectomy in the context of gastrectomy for gastric adenocarcinoma, we used direct comparison of outcomes with multiple regression to adjust for confounding factors. Given there were 150 patients in the D2 group compared to 54 in the D1/D1+ group, there is risk for outcomes to be confounded due to systemic differences between the two groups. In order to address this, we also performed propensity score matching, to estimate the causal effect of D2 lymphadenectomy compared to D1/D1+ and reduce the effect of bias, and furthermore compared the matched groups using a regression model to further adjust for imbalances.

## Conclusion

The Australian experience within this single unit shows that D2 lymphadenectomy with spleen and pancreas preservation can be performed safely on patients with gastric adenocarcinoma with excellent survival outcomes. Significant improvement in overall survival is observed in patients who underwent D2 lymphadenectomy, without increased surgical morbidity and mortality. This survival benefit was also seen in a subgroup analysis of N0 patients who underwent a D2 lymphadenectomy. This paper supports the notion of global consensus for D2 lymphadenectomy, particularly in the Western context.

## Abbreviations

AJCC: American Joint Committee on Cancer
BMI: Body mass index
Cum: Cumulative
DFS: Disease-free survival
KM: Kaplan-Meier
MAGIC: Medical Research Council Adjuvant Gastric Infusional Chemotherapy
OS: Overall survival
PSM: Propensity score matching
RFS: Recurrence-free survival
Surv: Survival
UICC: Union of International Cancer Control
Yr.: Year
